# Design and Application of Intelligent Reflecting Surface (IRS) for Beyond 5G Wireless Networks: A Review

**DOI:** 10.3390/s22072436

**Published:** 2022-03-22

**Authors:** Fred Chimzi Okogbaa, Qasim Zeeshan Ahmed, Fahd Ahmed Khan, Waqas Bin Abbas, Fuhu Che, Syed Ali Raza Zaidi, Temitope Alade

**Affiliations:** 1 School of Computing and Engineering, University of Huddersfield, Huddersfield HD1 3DH, UK; u2068709@unimail.hud.ac.uk (F.C.O.); w.abbas@hud.ac.uk (W.B.A.); fuhu.che@hud.ac.uk (F.C.); 2 School of Electrical Engineering and Computer Science, National University of Sciences and Technology, Islamabad 44000, Pakistan; fahd.ahmed@seecs.edu.pk; 3 School of Electronic and Electrical Engineering, University of Leeds, Leeds LS2 9JT, UK; s.a.zaidi@leeds.ac.uk; 4 Computer Science at the Department of Computing and Technology, Nottingham Trent University, Nottingham NG11 8NS, UK

**Keywords:** intelligent reflecting surfaces (IRS), mmWave, 5G networks, URLLC, IoT

## Abstract

The existing sub-6 GHz band is insufficient to support the bandwidth requirement of emerging data-rate-hungry applications and Internet of Things devices, requiring ultrareliable low latency communication (URLLC), thus making the migration to millimeter-wave (mmWave) bands inevitable. A notable disadvantage of a mmWave band is the significant losses suffered at higher frequencies that may not be overcome by novel optimization algorithms at the transmitter and receiver and thus result in a performance degradation. To address this, Intelligent Reflecting Surface (IRS) is a new technology capable of transforming the wireless channel from a highly probabilistic to a highly deterministic channel and as a result, overcome the significant losses experienced in the mmWave band. This paper aims to survey the design and applications of an IRS, a 2-dimensional (2D) passive metasurface with the ability to control the wireless propagation channel and thus achieve better spectral efficiency (SE) and energy efficiency (EE) to aid the fifth and beyond generation to deliver the required data rate to support current and emerging technologies. It is imperative that the future wireless technology evolves toward an intelligent software paradigm, and the IRS is expected to be a key enabler in achieving this task. This work provides a detailed survey of the IRS technology, limitations in the current research, and the related research opportunities and possible solutions.

## 1. Introduction

Existing wireless technologies lack the capacity to support emerging and future technologies such as artificial intelligence (AI) assisted communication [1], and Internet of Things (IoT) [2], and applications such as virtual reality (VR) [3], autonomous vehicles (AVs) [4], connected health [5], smart cities [6] and indoor localization [7]. The Cisco annual report published in February 2020 forecasts that by 2023, the fifth generation (5G) has to support 10 percent of mobile connections with an average speed of 575 megabits per second or a mobile connection speed which is 13 times faster than the current mobile connection speed and support approximately 14.7 billion machine to machine (M2M) type communication links (a 50 percent increase from 6.1 billion in 2018) [8]. Therefore, there is a need for an exponential increase in the capacity of present wireless systems to meet the desired throughput requirements to support future bandwidth-hungry applications [9]. The 5G and beyond mobile communication is expected to deliver an increase in spectral efficiency, user connection density and low latency communication [10]. After the completion of the 5G mobile communication standard (3GPP Release 15) in June 2018, the first mobile devices compatible with 5G were introduced to the market and the deployment of 5G supporting infrastructure commenced in most developed countries. The vision of 5G encompasses three use cases, namely: enhanced mobile broadband (eMBB), ultrareliable low latency communications (URLLC) and massive machine-type communications (MMTC) [11].

5G and beyond, like other generations of cellular technologies, demands a major paradigm shift in communications [12]. For high data-rate communication, 5G and beyond systems require massive bandwidth (up to 100×) more than the previous systems, high base station (BS) to support high device density and an unprecedented number of antennas at the BS [7]. However, for the requirements of IoTs and MMTC, the 5G and beyond technology needs to support high spectral efficiency, massive connectivity of users/devices, significantly low latency, short packet duration, low power, high mobility and diverse service types [13]. From the above two examples it can be observed that the 5G standardization does not prescribe a single enabling technology to support all the 5G and beyond application requirements [12]. Moreover, supporting technologies for 5G were reviewed over 10 years ago. Coupled with this, the rapid increase in the use of mobile IoT devices has created a challenge for wireless networks to meet the requirements of new applications, thus making it imperative that continuous effort is made to optimize energy efficiency (EE) and spectral efficiency (SE) to meet the data-rate and quality of service (QoS) of each user’s equipment [11,14,15,16].

Presently, sub-6 GHz bands are being utilized for wireless communications. To maximize the potential for 5G and beyond, the frequencies in the millimeter wave band, between the range of 10 GHz to 70 GHz, has been approved by the International Telecommunication Union (ITU) [12,17,18,19]. A 100-fold increase in the bandwidth is achievable by increasing the frequencies for transmission to the millimeter wave (mmWave) spectrum [20,21]. Until recently, mmWave was deemed unfit for mobile communications due to the hostile propagation qualities such as path loss, atmospheric and rain absorption, low diffraction and penetration through objects, strong phase noise and high cost [22]. The general notion for unlicensed bands around 60 GHz is for short-range transmission. At mmWave frequencies, objects and humans contribute to blockage and less diffraction occurs [20]. However, the miniaturization of semiconductors with reduced cost and power consumption has enabled us to surmount the limited range and propagation effects [23]. Moreover, specific frequencies suffer from absorption by gases with colliding resonance frequencies, such as 60 GHz for oxygen [12]. In addition, the radio environment in the current paradigm of wireless network optimization remains uncontrollable, and the propagated signal experiences reflection, diffraction, scattering and fading, which consequently becomes a limitation in maximizing the EE and SE of wireless networks [24].

One approach to overcome the poor propagation effects of the radio environment, especially in the mmWave band, is through the use of large antenna arrays with narrow beams to steer the beam energy coherently [12,19,20,25]. To overcome the large free space path loss in the mmWave band, the beamforming technique used in massive multiple input and multiple output (mMIMO) has been adopted for this purpose [26]. Beamforming techniques at 60 GHz, using mmWave integrated circuits, have shown support for wireless transmission of multi-Gb/s data, see [18,20,27,28] and references therein. Even with beamforming, the outdoor-to-indoor coverage is very limited in mmWave bands. With increasing frequency, rain causes a higher attenuation [12,19]. A newer technology i.e., intelligent reflecting surface (IRS) has the potential to overcome the poor propagation effects of the wireless channel and is fast becoming a household name in academia and within the industry due to the recent advances in the technology of metamaterials [29]. IRS is a novel hardware technology that can increase signal coverage and reduce energy consumption at a lower deployment cost [30]. IRS is also known as reconfigurable reflecting surface, large intelligent surface, and software-controlled metasurface. The advances in artificial electromagnetic materials has made the ability to control the communication environment through digital, programmable and reconfigurable methods using the IRS a reality [31].

An IRS offers a different solution to relaying and backscatter communications. An IRS is a metasurface consisting of many small reconfigurable passive low-cost reflecting elements that can easily introduce a controlled individual phase shift to the impinging electromagnetic wave and as a result alter the propagation characteristic between the transmitter and receiver to maximize signal strength and mitigate interference. The element in an IRS reflects incident electromagnetic waves independently with an adjustable phase-shift. In addition, as the IRS is passive, the incident signal is reflected without a dedicated energy source for RF processing, encoding/decoding and retransmission. Therefore, an IRS also offers an energy-saving solution when compared to amplify-and-forward (AF) relays as it only reflects the incident signals without any transmitter modulation [32]. Moreover, by smartly adjusting the phase-shifts of the elements using a smart controller, an IRS can employ beamforming to support dynamic wireless communication and suppress interference between users. These advantages have made the IRS a key technology for future wireless communications [30] and is the topic of this survey.

There are several surveys available in the literature on IRS technology. Ref. [33] discusses the applications of metasurfaces and distinguishes them from conventional frequency-selective surfaces. Authors in [34] provide an overview of IRS technology, including its main applications in wireless communication, competitive advantages over existing technologies, hardware architecture as well as the corresponding new signal model. In [35], authors highlight that the IRS network can only control the transmitter and the receiver and discusses path loss and asymptotic beamforming gain. Authors in [24] provide a survey on the IRS covering the basic concepts, performance metrics, reconfigurability and most recent applications. Ref. [36] discusses passive holographic MIMO surfaces that leverage the subwavelength metallic or dielectric scattering particles. Ref. [37] focuses on physical layer challenges for the implementation of IRS technology. In contrast to other surveys, the motivation of this survey is to provide a more comprehensive discussion on IRS technology with a focus on recent research advancements. Therefore, this survey provides a more detailed discussion on IRS technology and covers historical concepts, nomenclature, system architecture and design, an IRS-aided wireless network deployment and performance, smart radio channels with an an IRS and the limitations of the existing works and key future research directions. Furthermore, this survey covers several recent research advancements such as IRS-assisted UAV or UAV-satellite systems [38,39], an IRS aided for mobile edge computing [40,41], IRS-aided multiuser communication [42], IRS-aided localization [43,44], IRS-aided vehicular communications [45] and IRS-aided power transfer [46,47]. Finally, this survey discusses the current research gaps/opportunities and the possible solutions.

This survey discusses the design and application of an intelligent reflecting surface (IRS) for 5G and beyond wireless communication network. Due to the limitation of current systems and propagation losses in the mmWave band, the increased cost, complexity and power consumption resulting from increasing the number of transmit antennas with RF chains, an IRS is expected to provide the solution needed by offering better energy and spectrum efficiency to meet the desired throughput. The aim of this survey is to discuss the importance of an IRS in 5G wireless networks and highlight the potential of its use in future 6G and beyond wireless networks. Furthermore, this survey highlights key research opportunities/gaps covering a wide spectrum of areas of IRS technologys, i.e., channel estimation, beamforming design, practical protocol design, IRS-assisted heterogeneous networks (HetNets), IRS-aided unmanned aerial vehicles (UAVs), localization, device-to-device communication and future research direction to address those gaps. This paper builds on existing surveys by discussing the system architecture, providing a detailed overview of the internal architecture, performance limits and deployment strategy and also outlines potential scenarios of achieving a smart radio environment with an IRS.

**Figure 1:**
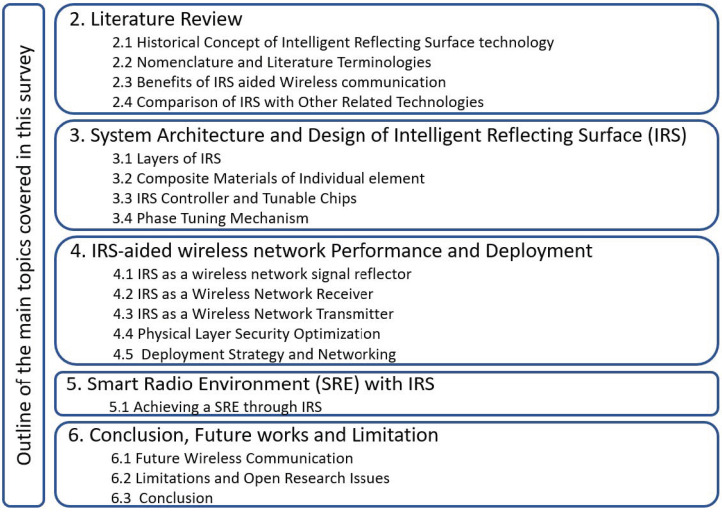
Outline of the covered topics.

The outline of this survey is shown in Figure 1, and is in the following order. In Section 2, a literature review is presented. In Section 3, system architectures of an IRS are presented. In Section 4, IRS-aided wireless network performance and deployment strategy is presented. In Section 5, a discussion on smart radio environment with an IRS is carried out. Finally, Section 6 provides a detailed discussion on the key research gaps/opportunities and the possible future research directions to address those gaps and also provides the conclusions.

## 2. Literature Review

This Section provides a summary of the existing literature in the context of IRS technology, nomenclature and literature terminologies and the-benefits of an IRS-aided wireless communication.

### 2.1. Historical Concept of Intelligent Reflecting Surface Technology

**Figure 2:**
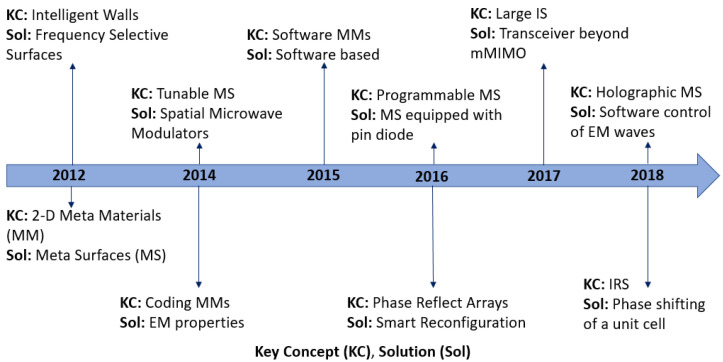
IRS timeline.

A summary of the historical perspective can be observed in Table 1 and Figure 2. Intelligent walls (IW), based on frequency selective surfaces (FSS), can alter the propagation direction, environment and performance of the electromagnetic (EM) waves through switching [48,49]. Subsequently, 2D metamaterials were designed to be an alternative to FSS for operation at various frequencies [33]. In [33], the authors outlined several use cases of 2D metasurfaces and discussed the ability of wave guides to trap and guide EM energy between two metasurfaces as well as the ability of terahertz devices to control metasurfaces to benefit operation at terahertz frequency. The benefits and applications in wireless communication of 2D metasurfaces, which occupy less physical space and experience lower loss when compared with three-dimensional (3D) surfaces, were also highlighted along with the operation at different frequency bands. Tunable metasurface-based spatial microwave modulators (SMM) were implemented by placing the SMM on the walls for maximizing the power or range of the transmission signal [50]. More recently, coding metamaterials (MM)s with the ability to manipulate the EM properties, replacing the phases 0 and π with a binary 0 and 1 [51] was introduced. This further helped in implementing the software program defined metamaterials as mentioned in [52]. Furthermore, programmable metasurface based on a PIN diode was introduced in [53] and references therein. Finally, in 2016 reconfigurable reflect arrays with passive elements were introduced, [54] and a large intelligent surface was proposed as a concept for beyond mMIMO [55,56,57]. In [58], the authors discussed backscatter principles and communication and reflective relay and introduced a large intelligent surface antenna (LISA). A software controlled hype-surface for IoT devices and for non-line-of-sight (NLoS) indoor communication was presented in [59,60]. Intelligent reflecting surface-based phase shifting of reflecting elements to steer the EM direction was proposed in [61].

### 2.2. Nomenclature and Literature Terminologies

IRS is the literature term adopted in this research paper, but the general idea refers to any passive intelligent or software-controlled metasurface with the ability to reconfigure the incident signal toward the user, thus creating a programmable wireless environment [11]. There are many common terminologies that refer to the same idea as an IRS. Some of them are highlighted in a recent survey paper [62]. IRS terminologies including those that are listed in [62] are mentioned below:

- Reconfigurable intelligent surface (RIS): It is a thin and cheap wallpaper-like surface that can reconfigure the radio propagation with the aid of a software program [63].
- Large intelligent metasurface (LIMS): These are equipped with a large number of low-cost metamaterial antennas with the ability to passively reflect the incident signals by certain phase shifts, without signal processing capability, thus improving the signal at receivers [64].
- Software defined metasurface (SDMS): It manipulates impinging EM waves in complex ways by altering the direction, power, frequency spectrum, polarity and phase, thus creating a programmable wireless environment [65].
- Large intelligent surface (LIS): It is aided by a large active antenna-array and dedicated RF chains to lower the power consumption of the system [56,66].
- Passive intelligent surface (PIS): This technology is an alternative to active antenna arrays and passively reflects incident signals, making it an emerging green technology. It can support the high data rate and energy sustainability demands of ubiquitously deployed users in 5G networks [67].
- Smart reflect arrays: These are closely related to an IRS with the capability to solve the problem of signal blockage in mmWave indoor communications by steering the incident signal toward the user destination to establish a robust link between transceivers [68].
- Passive holographic MIMO (HMIMO): It is a low-cost wireless planar surface composed of subwavelength dielectric scattering particles, that alters EM waves according to desired objectives and optimizes the wireless environment while achieving high-throughput, massively connected, and low-latency communications at a reduced power budget [36].

LIS, RIS, LIMS, SDMS and IRS are one and the same, and all these green technologies are expected to function similarly to improve the EE and SE at a lower cost. These do not perform any signal processing, rather they reconfigure the incident signal to the desired user, thus maximizing efficiency and are mostly based on phase shifting without amplification.

### 2.3. Benefits of IRS-Aided Wireless Communication

The IRS is expected to play a key role to meet the demand of the EE and SE of 5G and beyond wireless networks by optimally varying the reflection coefficient (i.e., phase shifts). An IRS performs passive signal reflection as it supports full-duplex communication without the need for radio frequency (RF) chains [42]. Moreover, an IRS is able to adapt the propagation environment by reconfiguring the reflection elements of the IRS, and as a result, the signal is beam-formed toward the receiver to enhance the desired signal and suppress multiuser (MU) interference [69].

The significance of a smart radio environment and the concept of uninterrupted connectivity where existing radio signals can be recycled through the design and deployment of an IRS and the potential of an IRS in future wireless networks (FWN)s is developed in [34]. Excited by the potential of a RIS, in [11], the authors discussed the historical perspective and differences with existing technologies, highlighted the theoretical performance limit of an IRS using mathematical techniques and discussed several fundamental research issues that need to be addressed and also elaborated the potential use cases in beyond 5G and networks. An overview of the IRS and its application, the advantage compared to similar technologies, design challenges and implementation of an IRS-assisted wireless communication network with basic numerical analysis is shown in [36,70]. In addition, [36] also presented a discussion about the HMIMO surface, a technology similar to the IRS which leverages on the subwavelength metallic or dielectric scattering particles. The concept of reconfigurability of an IRS, its most recent applications, performance metrics to characterize the improvement in IRS-assisted wireless networks and its practical challenges and effects on 5G and future wireless networks were highlighted in [24].

Some of the benefits of IRS-aided wireless communications are listed below

- Easy deployment and sustainable operation: An IRS is a 2D planar metasurface consisting of low-cost passive elements offering a high degree of freedom for many reflecting elements to be embedded on a single metasurface, thus making it easily deployable on buildings, walls, ceilings and underground tunnels with a clear line of sight (LoS) to the base station (BS). In addition, the absence of RF chains makes an IRS consume minimal power.
- Flexible reconfiguration via passive beamforming: Passive beamforming can be achieved by jointly optimizing the phase shift of each scattering element. Using the large number of reflecting elements, the incident signal can easily be directed toward the intended user and canceled in other directions, thus improving the overall performance gain of the wireless network [24].
- Dense deployment: To provide higher data rates and also due to the limitation of transmission range of mmWave bands, 5G is required to be a dense network. However, a dense BS deployment causes a significant increase in interference, resulting in a lower signal-to-interference plus noise ratio (SINR) and thus, a lower throughput. An IRS is extremely useful in such a scenario because they can be used to increase the signal power and reduce the interference power at the receiver through smart beamforming and enhance the system capacity with low implementation cost.
- Reduced cell edge outage: At the cell edge, users experience lower signal power and higher interference. Again, in this case, by suppressing interference, an IRS can improve the overall signal quality for the cell-edge users. The scattering elements can split the signal and assist data in MU wireless networks. Thus, an IRS improves the sum-rate performance and delivers better QoS with reduced energy consumption.
- Support emerging technologies: In emerging technologies such as virtual reality (VR), holographic communication and other IoT applications, an IRS will be an essential element to fulfill their very high data rate requirement [24].
- Applications: The key applications of the IRS are in the area of non-line-of-sight (NLoS) transmission and blockages, smart wireless power transfer, enhanced security, interference cancellation, etc., by intelligently controlling the signal propagation.

Summary of literature survey can be observed in Table 2.

### 2.4. Comparison of IRS with Other Related Technologies

An IRS has several distinct feature that distinguish it from other technologies. Some of them are listed below:

- IRSs are passive metasurfaces with the ability to reflect incident signals without the use of a dedicated energy source.
- IRSs do not require analog-to-digital converters (ADC)s and digital-to-analog converters (DAC)s and power amplifiers to amplify or introduce noise when reflecting signals and thus provide an energy efficient solution.
- IRSs are easily deployed on walls, ceilings, etc., in an indoor environment due to their transverse size.
- Full band response makes it possible for an IRS to operate at any frequency, and they support full duplex transmission.

Compared to other closely related existing technologies that are presently used in wireless networks such as mMIMO, AF and Decode and Forward (DF) relay, and backscatter communications [63,76,77,78], an IRS offers a totally different solution and hence, a competitive advantage. Comparison of an IRS with closely related technologies is summarized in Table 3.

- IRS vs. mMIMO: The IRS is different from the active intelligent surface-based massive MIMO due to their different array architectures (passive versus active) and operating mechanisms (reflect versus transmit).
- IRS vs. AF Relay: AF relays play a role in source-destination transmission by amplifying and regenerating the signals whereas, an IRS reflects the incident signals as a passive array without the use of a transmitter, thus eliminating the need for transmit power consumption. An IRS is expected to function in full duplex mode while AF operates in half duplex mode as it suffers from severe interference in full duplex mode which makes it require effective interference cancellation techniques, thus making IRS more spectral efficient.
- IRS vs. DF Relay: Similar to AF relaying, DF relaying decodes and regenerates the transmitted signal from the source and transmits it to the destination. Due to the decoding operation, it has a much higher complexity and consumes high signal processing power [79]. In contrast, as mentioned previously, the IRS does not perform any decoding and only performs passive reflection. Thus, it has a lower cost and consumes negligible power.
- IRS vs. Backscatter communication: Backscatter communication reflects an ambient radio frequency identifier (RFID) tag to the receiver from the signal sent by the reader. The IRS improves the existing communication link performance instead of delivering its own information by simple reflection of the signal. As such, the path from reader to receiver in backscatter communication experiences undesired interference and needs to be canceled/suppressed at the receiver. However, in IRS-aided communication, both the direct-path and the reflect-path signals carry the same useful information and can be constructively added at the receiver to maximize the total received power.

## 3. System Architecture and Design of Intelligent Reflecting Surface (IRS)

The growth of EM-MMs has been tremendous in recent times due to their ability to manipulate the EM waves in unconventional ways, leading to phase shift, amplitude modulation (AM) and polarization conversion of EM waves [80]. Coding digital and programmable MMs are realized by simplified design and optimized phase reflection coefficient, thus realizing a simple and an efficient way to manipulate the EM waves for various application including mmWave and terahertz frequencies which can be independently controlled by a field-programmable gate array (FPGA) program, thus creating a link between the physical and digital world [51,80].

**Figure 3:**
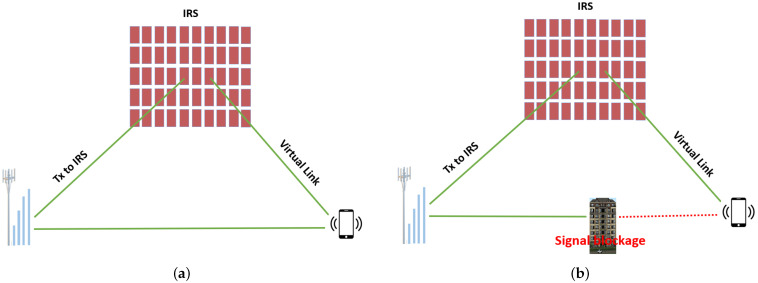
IRS-aided wireless transmission system: (**a**) In the presence of LoSlLink; (**b**) In the absence of LoS link.

Figure 3 shows a MS consisting of a large array of passive elements with the ability to transform impinging EM waves to several directions in real time [81]. In Figure 3a, there is a clear LoS between the BS and the smartphone. Therefore, the presence of IRS will enable the generation of a replica copy and result in improved diversity of the system. While in Figure 3b, the LoS is blocked due to the presence of the high rise building, the IRS will help establish a virtual link between the BS and the smartphone. This virtual link will enable the receiver to achieve the transmitted signal, thus improving the overall EE and SE of the system, which was not possible without the presence of the IRS.

### 3.1. Layers of IRS

**Figure 4:**
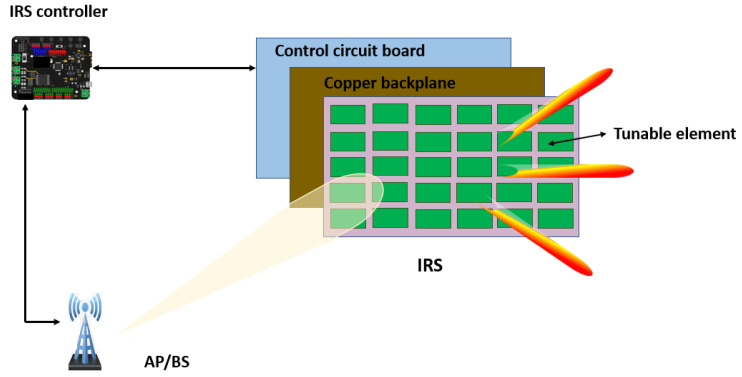
Architecture of an IRS.

A typical IRS has three layers as shown in Figure 4. These layers can be explained as follows:

- The first/outer layer: This layer consists of a large array of passive reconfigurable patches printed on a dielectric substrate to manipulate the incident signals [81].
- The second/intermediate layer: This layer consist of a copper plate to reduce signal energy leakages during reflection [81].
- The third/inner layer: This layer consists of a control circuit board with the ability to steer the reflection phase and amplitude in real time. The smart controller is typically a FPGA which regulates the reflection and configuration and also serves as a gateway between the BS and the destination [81].

### 3.2. Composite Materials of Individual Element

An IRS comprises of a large 2D artificial array of passive reconfigurable dielectric patches printed on a dielectric substrate possessing the ability to manipulate the direction of incident signals. An IRS is composed of a large number of low-cost components such as varactors and diodes [82]. A unit cell patch is modeled independently as a passive scattering element patch. The intercell distance is in the order of a wavelength, usually half a wavelength or five to ten times smaller than the wavelength [34].

### 3.3. IRS Controller and Tunable Chips

The individual elements of an IRS can be reconfigured electronically with the aid of devices such as positive-intrinsic-negative (PIN) diodes, field-effect transistors (FETs) or micro-electromechanical system (MEMS) switches [80,83], which are commonly used due to their fast reflection response, low reflection loss, low energy and hardware cost [81]. Generally, in an IRS the equivalent PIN diode circuit is employed to control the biasing voltage with the help of a direct current (DC) feeding line. The PIN diode has two stages: ON or OFF. The stages can be generated with the help of a phase-shift difference of π [51]. The different phase shifts of the individual elements of an IRS can be obtained by varying the corresponding biasing voltage of the smart controller to adjust the reflection amplitude. A variable resistor load may be applied to each element design as mentioned in [84]. Practically, it is preferred to have an independent control of the amplitude and phase shift of each element followed by an efficient integration of the IRS circuit [84]. An IRS is able to reconfigure incident signals. This helps in reducing the intercell communication because of the tunable chips [24]. Communication between the IRS unit cells can be either wired or wireless, with the former being preferred, and the interconnect can easily cointegrate controllers on the same chip as implemented in low-power embedded systems. Wired intercell communication for the IRS can be limited when scaling the size. Multiple chips can result in a complex layout and routing. Considering energy, latency and robustness, it is ideal to implement wireless intercell communication for larger-scale or dense IRS [85].

### 3.4. Phase Tuning Mechanism

The incident signals are steered toward the user by manipulating the phase of each unit element of the IRS [24]. Initially, MSs were designed for fixed tasks at specific frequencies and required a redesign and refabrication to operate at a different frequency range. However, presently MSs have evolved to a greater extent, and they are able to respond to a software-controlled stimuli. This software-controlled stimuli will be able to adjust the EM waves toward a desired direction without requiring a redesign of the tuning mechanism for different frequency or functionality [86,87]. External stimuli such as electric, magnetic, light or thermal stimuli when applied induce a change in the MS functionality that can be referred to as the global tuning mechanism [88]. Local tuning occurs at the unit cell by integrating biased diodes into the individual unit cell elements to turn unit cells locally and enable them to reconfigure and modify functionality [85]. Moreover, electrically controlled binary-phased tuning or continuous reactant mechanisms by using diodes or varactors is a reliable tuning approach [89]. An IRS has a a wide range of tunable functions such as perfect absorption, anomalous reflection, beam shaping and beam steering [24].

## 4. IRS-Aided Wireless Network Performance and Deployment

An IRS improves the performance of a wireless network by transforming the propagation channel into a controllable radio environment, thereby realizing better SE and EE [12]. The ability to control the radio environments changes the design and optimization of wireless networks. Manipulation of the EM waves with the use of software overcomes the highly probabilistic nature of EM propagation. An IRS can prevent the effect of multipath fading by coherently combining the reflected, refracted, and scattered radio waves at the desired destination [90].

### 4.1. IRS as a Wireless Network Signal Reflector

An IRS can be employed as a signal reflector. In [68], the authors established that an IRS can reflect the incident signal toward the receiver to establish a reliable virtual link between transceivers and thus overcome the NLoS challenges in mmWave communication. The benefits of using an IRS as a signal reflector can be quantified using two types of performance metrics: probabilistic metric and ergodic metric. Next we discuss these metrics in detail.

#### 4.1.1. Probabilistic Metrics

The probabilistic metric can help depict the uncertainty in the quality of wireless transmissions of individual transceivers [91]. An analytic approach to derive the probability of an IRS when it acts as a reflector was proposed in [92]. It was shown that whenever a surface is coated with a MS, any angle of incident can be used to synthesize an angle of reflection [92].

#### 4.1.2. Ergodic Metrics

The ergodic metric determines the average network performance [24]. Ref. [93] showed that the data rate between the BS and a mobile user can be improved even if the design of the IRS’s optimal phase shifts is dependent on limited feedback from the mobile user. An IRS with perfect channel state information (CSI) reduces positioning error bound and orientation error bound [94]. Moreover, when the direct path channel between the transceivers is characterized by a low rank matrix, through optimization an IRS can improve the rank of channel matrix and lead to significant capacity gains [95].

#### 4.1.3. Throughput Performance of IRS

**Figure 5:**
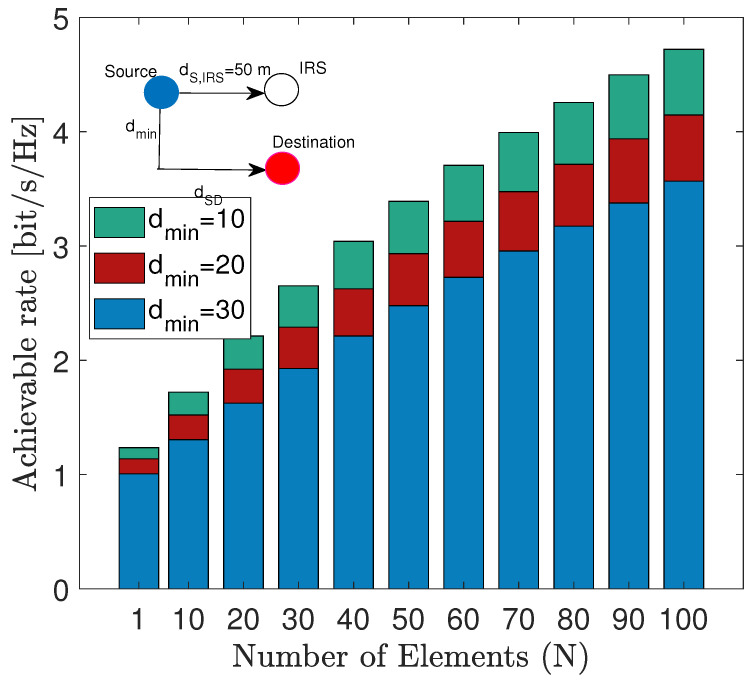
Throughput of IRS against varying number of elements and minimum separation between source and destination.

Consider a link between an arbitrary source and its intended destination which is blocked by a temporary or permanent obstacle. The link is therefore established using an IRS with *N*elements. The channel gain between the source and destination is given by $h_{s,d}$, the source and IRS is given by $h_{s,IRS}\in C^{N}$ and similarly the channel gain between the IRS and destination is given by $h_{IRS,d}\in C^{N}$ then as shown in [69] the achievable rate is given by:

(1) $$R=\log_{2}\left(1+\frac{P_{TX}\left(\sqrt{\beta_{sd}}+N\alpha \sqrt{\beta_{IRS}}\right)}{\sigma^{2}}\right),$$

where $P_{TX}$ is transmit power, $\beta_{sd}={\left|h_{sd}\right|}^{2}$, $\beta_{IRS}=\frac{1}{N}\sum_{n=1}^{N}{\left[h_{s,IRS}\right]}_{n}{\left[h_{IRS,d}\right]}_{n}$, α∈(0,1] is amplitude of reflection associated with the IRS and $\sigma^{2}$ is noise variance. Furthermore, let us assume that the LoS and NLoS propogation follows as articulated in [69], then Figure 5 shows the achievable rate against minimum vertical separation between the IRS source and the destination. As shown in Figure 5 the throughput decreases with an increase in separation as the path loss increases with the distance. Moreover, the throughput increases with an increase in the number of elements as expected. The interesting insight here is that the decrease with distance is more pronounced when *N* is small. This implies that perhaps an adaptive selection of *N* which depends on the distance between source and destination should be employed. As a matter of fact, such distance dependent adaptations are common features of current wireless networking standards. In particular, distance dependent adaptive rate selection is part of most 802.11x standards. Therefore, it is plausible to explore distance dependent activation of *N* to realize a similar rate adaptation technique for IRS empowered future wireless networks.

### 4.2. IRS as a Wireless Network Receiver

An IRS can be used as a signal receiver by deploying antenna arrays in the form of an IRS to improve the overall coverage of a wireless network [56]. An IRS was employed as a signal receiver for communication beyond mMIMO in [96]. In this paper [96], the BS was designed as a radiating surface instead of the traditional MIMO BS with active antenna arrays. This approach will help reduce the power and hardware impairments.

### 4.3. IRS as a Wireless Network Transmitter

An IRS can also serve as a transmitter. For low-cost transceiver architectures of 5G and beyond, employing spatial modulation (SM), a promising concept of index modulation (IM), can achieve good SE at a low complexity with a trade-off of error performance [97].

### 4.4. Physical Layer Security Optimization

Physical layer security of a wireless communication channel in the presence of an eavesdropper can be evaluated using a basic wiretap model [98]. An IRS can enhance physical layer security in wireless communications by ensuring a lower wiretap transmission rate than the secrecy capacity of the channel [99,100]. MIMO beamforming techniques improve the security by suppressing the signal-to-noise-ratio (SNR) of the eavesdropper and enhancing the SNR of the legitimate receiver [101]. Similarly, deployment of an IRS, in any wireless network having eavesdroppers, can improve the security of the network by jointly optimizing the beamformers at the BS and IRS reflection coefficients [100].

### 4.5. Deployment Strategy and Networking

As discussed previously, an IRS when deployed in a wireless network improves the network performance by reflecting the incident signals. However, the overall network performance depends on the deployment strategy used in the wireless network. Deployment strategy includes factors such as deployment locations, size of the IRS, user distribution and demand, cost of deployment, operational cost, availability of space and the propagation environment [81]. IRS deployment strategy is completely different from the deployment strategy used for active communication nodes such as APs, BS and relays. Unlike an IRS, active BSs and APS are spaced geographically to achieve maximum coverage while active relays are deployed between the transmitter and receiver to balance the SNR of the two-hop link. It is typically ideal to deploy an IRS in the close vicinity of the BS and users since IRSs are passive (only reflect and lack amplification and signal processing power). Moreover, due to the low cost, power and negligible interference of an IRS, it can be densely deployed in a wireless network and spaced sufficiently since its signal power rapidly decays with distance [81]. An IRS can be deployed in a distributed or centralized manner. Two IRS deployment strategies widely discussed for a multiuser scenarios based on the number of reflecting coefficients are briefed below [81].

- Distributed IRS deployment strategy [102]: The IRSs are densely deployed around one BS in a wireless network to serve multiple user clusters. The achievable rate is reduced since the user receives passive beamforming gain from only the closest IRS due to significant distance from the other deployed IRSs.
- Centralized IRS deployment Strategy [42]: All IRS reflection coefficients are kept central to the BS or AP in one location. The beamforming gain in this scenario will be larger due to more IRS reflecting elements. However, the gain may be reduced as the number of served users increases.

These deployment strategies have varying achievable rates in different scenarios, and it is difficult to choose a single best strategy [42].

## 5. Smart Radio Environment (SRE) with IRS

Smart radio environment is an emerging paradigm shift from limitation of uncontrollable propagation of radio signals in the existing radio environment. Presently, the optimization of wireless networks is performed at the communication endpoints, i.e., transmitters and receivers. Until now, several advanced techniques such as modulation/encoding schemes and multiple antenna processing at the transceivers, efficient transmission and retransmission protocols, and robust demodulation and decoding methods at the receiver have been proposed to improve the performance of wireless networks [78]. The wireless environment, on the other hand, has remained largely uncontrollable, making communication engineers model transceivers to adapt to the propagation environment.

### 5.1. Achieving a SRE through IRS

IRS is a revolutionary technology with the potential to provide efficient use of spectrum in a radio environment by the software defined control of the propagating signals and to meet the requirement of emerging and future technologies [59]. Present wireless networks are designed and optimized at both the transmitter and receiver to compensate for certain effects of wireless channels. An IRS is essential in designing a programmable wireless environment as opposed to traditional uncontrollable wireless channels and thus serve a vital role in the sixth generation (6G) network [103]. The concept of SRE goes beyond reflecting incident signals and supports a wide range of applications such as reducing interference, enhancing security, and extending the range of communication to mention a few. To meet the requirement of SRE, wireless channels should be taken into account as part of the network design that needs to be optimized to support diverse performance metrics and quality of service requirements [78].

#### 5.1.1. Use Cases of IRS for Future SRE

Three main use cases of the IRS for future SRE are discussed below:

- Coverage enhancement: By deploying an IRS in a wireless network, a virtual link can be created to overcome NLOS conditions to deliver better connectivity to the desired user.
- Interference suppression: An IRS can enhance the SINR by suppressing unwanted signals that may interfere with users in a communication network.
- Enhanced security: An IRS can create destructive interference at eavesdroppers or steer the signals to directions not occupied by unintended users and as a result, enhance the security.
- Indoor localization: An IRS can improve estimation of the location of mobile terminals and devices to achieve reliable radio localization and mapping.
- Information and power transfer: Can be achieved by using an IRS to get ambient EM waves and concentrating them toward low-power IoT devices and sensors to simultaneously transfer wireless information and recharge low power sensors and IoT devices [36].

#### 5.1.2. Potential Concepts for SRE

Based on the discussed use cases, SRE can support the following potential concepts:

- Smart cities: An IRS with large transverse sizes can be used to coat the facades of building in cities to increase spectral efficiency, enhance coverage and reduce the exposure to EM radiation in outdoor environments by replacing most BS infrastructures with IRS.
- Smart clothing: Embedding smart sensors and metamaterials into clothing can create wearable body networks for monitoring the health of people.
- Smart homes: The interior walls or ceilings at home can be coated with sizeable IRSs to enhance the local connectivity of devices (mobile phones, tablets, IoT devices etc.).
- Smart buildings: In buildings, large windows can be replaced with low-cost IRSs to achieve a better indoor to outdoor communication.
- Smart malls: IRSs can be coated within a mall that will improve spectral efficiency and provide the necessary connectivity, shop map and localization information to a large number of users simultaneously.
- Smart hospitals: By deploying an IRS in hospitals, local coverage can be enhanced without the need of increasing the transmitted power and enhance the local connectivity of IoT devices.
- Smart factories: An IRS can improve the performance of smart factories by enhancing the coverage and the transmission rate to support efficient low latency machine-type communication.
- Smart university campuses: An IRS can improve the connectivity within a university campus by coating the exterior of large buildings, and indoors of offices, classrooms delivering reliable connection around the campus.
- Smart airport: In airports, large numbers of users may take different directions when disembarking from airplanes. IRSs can be employed for steering different beams toward different hallways to enhance the quality of the received signals. Also, IRSs may be used to enhance the signals in high-speed download areas, e.g., Internet areas in close proximity of the gates.
- Smart Stadiums: In stadiums by deploying several IRSs, wireless network capacity can be maximized to provide the necessary connectivity to serve multiple devices of large numbers of users simultaneously.
- Smart train station: In train stations, several users may be waiting on the platforms before the arrival of trains. IRSs can be deployed for illuminating areas of the platforms r to enhance the received signals of individual users or clusters of users.
- Smart underground car parks and tunnels: An IRS can be fitted in a location with clear LoS to the BS and serve users in undergrounds car parks and tunnels and provide localized information for a guiding map application.
- Smart cars: Cars can be coated with IRSs to provide reliable communications within the car itself, as well as serving as enhanced vehicle-to-vehicle and vehicle-to-infrastructure communications.
- Smart trains: The interior of trains may be coated with IRSs to provide a better signal coverage for passengers and reduce the levels of EM exposure of passengers.

## 6. Conclusions, Future Works and Limitation

The development of technologies such as artificial intelligence, virtual reality (VR), UAV and smart cities that require higher bandwidth to support connectivity has created the necessity for wireless networks to possess the capacity to offer seamless connectivity to support these applications. The fifth generation is expected to deliver eMBB, URLLC and mMTC, and the sub-6GHz band is insufficient for achieving this.

Therefore, the ITU approved the use of mmWave frequency band for mobile communication making it possible to achieve the bandwidth required. However, the frequencies in the mmWave band suffer a lot of propagation effect and fading and a further increase in antenna array optimization at the transceiver in order to compensate for these losses. This consequently leads to additional complexity, cost and interference issues. Recent advancements in metasurfaces have created an opportunity for the wireless channel to be controlled by software and computer programs. The IRS is a passive 2D metasurface with the ability to smartly reflect the EM waves from the source to the destination and thus smartly control and reconfigure the wireless channel.

This survey gave an overview of the historical perspective on the IRS, outlined its distinct features, and discussed in detail the benefits of IRS-aided wireless communication for present and future applications. It was demonstrated that an IRS has a competitive advantage despite being a new technology when compared with closely related technologies such as AF-relays and DF-relays in terms of spectrum, power and energy efficiency.

The system architecture of an IRS was illustrated discussing the composite materials and internal architecture mechanisms. The performance of an IRS-aided wireless network was discussed explaining the probabilistic and ergodic metrics. In addition, various use cases of an IRS in wireless communications such as a signal reflector, signal receiver and signal transmitter were presented. These demonstrated the ability for an IRS to be deployed in diverse ways in the future and can potentially change the architecture of the base station as well as the cellular network.

Finally, the survey presented an opportunity for an IRS to facilitate the emergence of a smart radio environment by the joint optimization of the transmitter, receiver and wireless channel. This smart radio environment puts an end to the understanding that the wireless channel is uncontrollable and therefore wireless networks can only be optimized at the transceiver ends. A summary of the challenges and their suggested solutions is given in Table 4.

### 6.1. Future Wireless Communication

Recent research in the areas of IRS-aided UAVs, mobile cell edge computing, mmWave and THz communication and D2D communication has shown that the IRS will play a vital role in the future of wireless communication. However, the literature in these areas is still in its infancy and further research is required to address issues such as how to maximize the achievable secrecy rates, how to reduce learning error in M- based solutions and how to enable reliable THz communication. The following text discusses these aspects in detail.

- IRS-aided UAV: IRS mounted on UAVs or on buildings can significantly improve connectivity between the UAVs and the connection node, making it more reliable for mission critical operation. A secured IRS-assisted UAV system with the aim of maximizing the average secrecy rate is proposed in [38]. As generally the problem is nonconvex and nondeterministic polynomial-time (NP) hard, it is difficult to find an optimal solution. Refs. [38,110,111] and references therein propose iterative solutions to tackle this problem. Furthermore, RIS-assisted integrated UAV-satellite communication for terrestrial networks has also been recently explored and is an active area for future research [39].
- IRS-aided mobile cell edge computing (MEC): An IRS when deployed for MEC, can assist to offload the computational load onto the BS to reduce latency and energy consumption of the computing device [41]. Ref. [40] proposes ML tasks at the MEC server with the assistance of an IRS. The main focus is to minimize the maximum learning error of all the participating users. In cities the MEC performance is dependent on scheduling and offloading conditions [116]. Therefore, designing task scheduling algorithms to improve resource allocation will be a key challenge.
- IRS-aided mmWave and THz communication: The IRS is envisioned to be a key player is 5G mmWave systems and future 6G terahertz systems by compensating for the losses these higher frequencies suffer due to blockages and the distance between the transmitter and the receiver. An IRS deployed in future wireless networks can potentially save costs and reduce reliance on multiple antennas at the BS. Channel estimation and beamforming design for mm-Wave and THz will be quite a challenge and will generate an exciting area for future study.
- IRS-aided D2D communication: IRS is expected to assist D2D communication by improving the communication links between devices by providing a robust virtual link in case of blockage. However, when offloading using D2D devices latency [112,113], sum-rate maximization [114] and secrecy-rate maximization [115] will be key issues. Developing and designing D2D algorithms to solve these abovementioned issues will be an important area of research.
- IRS-aided vehicular communications: An IRS can be combined with short-range as well as long-term evolution (LTE) to provide cellular communications for vehicles and infrastructure [45]. However, as the movement of the vehicles in the city centre will be slow, designing an IRS-based solution will require very thin pointed beams. However, the beamforming will require exact and complete CSI which as described earlier is a very difficult task, and an AI/ML based solution might be developed for vehicular communications using an IRS.
- IRS-aided localization: An IRS could be employed for localized application, especially for indoors. The indoor environment is difficult to simulate due to different scenarios such as offices, malls, train stations, cinemas, houses, etc. Furthermore, multipaths due to wider band create an environment in which the first arriving paths do not contain the maximum energy. An IRS could be employed as a self-sensing architecture that could help determine the departure and arrival angles by employing the MUSIC, ESPIRIT, maximum likelihood, etc., algorithms [43,44]. An IRS-aided localization for indoor communications could resolve the object within centimetres which is not currently possible with BLE or WiFi standards.
- IRS-aided power transfer: Directing the signal power accurately toward a specific location requires perfect CSI. However, perfect CSI is not available at the IRS. Therefore, the IRS has to utilize imperfect or incomplete CSI to calculate the phases for signal reflection. Due to imperfection in the CSI, the reflected signal power toward the desired transceiver is reduced. This spread of power also increases the interference to the other transceivers in the region [46,47]. Thus, it lowers the overall throughput of the network.

### 6.2. Limitations and Open Research Issues

Although there has been extensive research in the area of IRS-enabled wireless communication, there are key areas such as energy efficient channel estimation, practical protocols for information exchange, reflection as a source of IRS-assisted HetNets and flexible lightweight phase reconfiguration that require further investigation. These aspects are discussed below.

- Energy efficient channel estimation: The ability for an IRS to accurately direct the incident EM wave to the receiver while maintaining the passive attribute is vital in the design of an IRS. The signal processing for IRS-aided wireless communication is expected to be performed at the BS, thus requiring the IRS to sense the channel and steer the EM waves accordingly [24]. To implement the abovementioned future wireless communication systems, the core requirement is to have a complete CSI. However, exact CSI is difficult to obtain, therefore, long-term and/or near-instantaneous CSI could be considered. However, due to the large number of reflecting elements, the channel matrix is going to be too large and will require many pilots for training. Therefore, designing an efficient training sequence which could reduce the overhead would be a future challenge when employing an IRS for near-instantaneous CSI estimation. Furthermore, for long-term estimation we might require the angle or location information which might be a key challenge for indoor communications especially in a NLOS scenario. This will further lead to mobility challenges, especially when we are trying to track the movement of a fast-moving individual or an object. Both the near-instantaneous and long-term CSI estimation require several key parameters to be estimated as well as many iterations to find an optimal solution if the problem is convex or a model-based optimization methods for nonconvex problems. ML/AI-driven solutions may be sought as for this issue. One option is to employ ML/AL-based data driven solutions where feature extractions without mathematical models can be carried out. It has been found to have quite robust solutions in imperfect CSI and hardware implementation in other fields. Therefore, it might be another important and timely approach for IRS-based systems in the future.
- Beamforming design: Considering various degrees of CSI availability, namely imperfect CSI, partial CSI and statistical CSI, the beamforming design must be varied to keep the interference to a minimum level and enable robust communication [105,106]. Codebook-based beamforming may also be utilized to reduce the training overhead, reduce the interference and improve reliable/robust communication. These codebook-based methods require the codebook to be filled up overtime with relevant CSI data. The varying channel state information will make the codebook outdated and will require better prediction methods to forecast the future CSI based on existing codebook data.
- Practical protocols for information exchange: In designing information exchange protocols, factors such as low power consumption and latency should be considered. Particularly, MAC and joint layer optimization solutions should be addressed. Most of the proposed work carried out in the IRS domain is along the physical layer. However, MAC layer and joint optimization of physical and MAC layer still are open questions. Furthermore, the problem is exacerbated by the limited number of available datasets that can be employed for joint optimization of these layers.
- Reflection as a source of IRS-assisted HetNets: An IRS offers a green technology alternative to existing systems and is expected to significantly improve future wireless systems. Another challenge is that the massive deployment of IRS to form HetNets would require optimized techniques to serve multiple data streams. A centralized coordination for the IRS-assisted HetNets and transceivers need continuous channel training for learning the CSI [44,109].
- Flexible lightweight phase reconfiguration: The challenge of CSI increases as the transverse size of the IRS increases. Putting cost and computational complexity into account, it is important that flexible and low-complexity algorithms are designed for an IRS.

### 6.3. Conclusions

This paper presented a comprehensive survey on the design of the IRS as a promising technology for 5G wireless networks and provides a detailed overview on several IRS applications in wireless communication. The paper built upon the existing knowledge that demonstrated the importance of IRS in the design of a wireless communication network. Highlighting the IRS as a key technology in achieving a smart radio environment demonstrated the potential of an IRS to aid in the joint optimization of the transmitter, receiver and propagation channel. Particularly, this survey provided a detailed discussion on the IRS technology covering its system architecture, internal architecture, performance limits and deployment strategies and also highlighted the potential scenarios that can benefit from a smart radio environment. Furthermore, key limitations, open research opportunities and future directions have also been discussed.

## Figures and Tables

**Table 1:** Historical Perspective of IRS.

Key Concepts	Solution	References	Year
Intelligent Walls	Frequency Selective Surfaces	[48,49]	2012
2-D Meta Materials (MM)	Meta-Surface (MS)	[33]	2012
Tunable MS	Spatial Microwave Modulators	[50]	2014
Coding MMs	EM properties	[51]	2014
Software MMs	Software based	[52]	2015
Programmable MS	MS equipped with pin diode	[54]	2016
Phase Reflect Arrays	Smart Reconfiguration	[53]	2016
Large IS	Transceiver beyond mMIMO	[36,55,56]	2017
Holographic MS	Software control of EM waves	[59,60]	2018
IRS	Phase shifting of unit cell	[61]	2018

**Table 2:** Related Works on the Review on IRS Technology.

Reference	Contribution
[11,34,36]	Application and potential use case of an IRS in 5G and FWNs
[33]	Application of 2D MS for controllable smart surfaces and EM at various frequency bands.
[34]	Theory and the design of an IRS to achieve a smart radio environment and further discussions on the deployment in FWNs.
[11]	Theoretical performance limit of an IRS using mathematical techniques and the discussion of fundamental research issues needed to be addressed and elaborates the potential use cases in FWNs.
[36]	Discussion on HMIMO surfaces, a technology similar to IRS which leverages on the subwavelength metallic or dielectric scattering particles.
[70]	Discussion on the overview of the IRS, advantage when compared to similar technologies, design challenges and implementation of IRS-assisted FWNs.
[58]	Discussions on backscatter principles and communication, reflective relay and introduction to large intelligent surface/Antenna (LISA).
[24]	Survey on IRS, highlighting the basic concept of IRS. Reconfigurability and its most recent applications and performance metrics to characterize the improvement in IRS-assisted FWNs.
[71,72]	Application of a HS approach to achieving a programmable control over the behavior of a FWN.
[35]	Review of Reconfigurable Intelligent Surface Myth and reality.
[73]	IRS enhanced OFDMA system is proposed.
[37]	Challenges and Opportunities of an IRS in FWNs.
[74]	Amalgamate IRS and relay to improve the system performance of FWNs.
[75]	Buffer aided relays to enhance system secrecy rate with a delay constraint.

**Table 3:** Comparison of IRS with Other Related Technologies.

Technology	Role	Duplex Mode	Power Budget	Noise	Interference	Hardware Cost	Energy Utility
IRS	Helper	Full	Passive Low	No	Very Low	Low	Low
AF Relay	Helper	Half	Active High	Additive	High	High	High
DF Relay	Helper	Full	Active High	Additive	High	High	High
Back-Scatter	Source	Full	Active Low	Additive	Low	Low	Very Low
mMIMO	Source	Full	Active Very High	Additive	High	High	Very High

**Table 4:** Opportunities/Challenges of IRS Technology and Future Research directions.

Challenge/Opportunity	Description	Future Directions
Channel estimation algorithms [24,47,104]	With large antenna arrays, accurate channel estimation has practical limitations.	Design and develop EE and long term or near instantaneous CSI estimation algorithms. ML/AI driven solution that has shown robustness against imperfect CSI.
Beamforming design [45,105,106,107]	It is dependent on the accuracy of the estimated CSI. However, the beamforming solution can be different depending upon the application.	Three different approaches such as Codebook based solutions considering fastly varying CSI ML/AI based solution to forecast the future CSI based on the existing codebook data. For vehicular communication, the goal is to reduce the latency when designing the narrow beams.
Practical protocol design [40,41,108]	Most of the work on IRS is focused on the physical layer. Practical MAC layer and/or joint physical and MAC layer protocol design is an open area for research.	For MAC layer Designing task scheduling algorithms to improve resource allocation in MEC will be an exciting research area. Designing joint optimizing algorithms/solutions for MAC and PHY layer. to reduce latency and improve secrecy when employing an IRS.
IRS assisted HetNets [44,109]	Massive deployment of IRS to form HetNets. Energy efficient solution will be a key requirement.	To develop optimized techniques with a focus to serve multiple data streams for IRS-assisted HetNets. To investigate the performance of IRS-assisted HetNets with a focus on energy efficiency by incorporating hardware impairments
IRS-aided UAVs and D2D Communication [38,39,110,111,112,113,114,115]	IRS can assist UAVs and D2D communication even in the presence of blockages.	To develop and design algorithms To reduce latency, and/or To maximize sum rate and/or secrecy rates or jointly optimizing them are active areas for research in the future.
IRS-aided localization [43,44]	IRS technology can assist localization, particularly indoor, by providing better control of the environment.	To devise algorithms that can provide better estimate of AOD and AOA that would enable localization within centimeter level accuracy.

## Data Availability

Not applicable.

## Abbreviations

The following abbreviations are used in this manuscript:

2D	Two dimensional
3D	Three dimensional
5G	Fifth Generation
ADC	Analog-to-Digital converters
AF	Amplify-and-Forward
AI	Artificial Intelligence
AM	Amplitude Modulation
AOA	Angle of Arrival
AOD	Angle of Departure
AV	Autonomous Vehicles
BS	Base Station
CSI	Channel State Information
DAC	Digital-to-Analog converter
DC	Direct Current
DF	Decode and Forward
EE	Energy Efficiency
EM	Electromagnetic
eMBB	enhanced Mobile BroadBand
FET	Field-Effect Transistors
FPGA	Field Programmable Gate Array
FSS	Frequency Selective Surfaces
FWN	Future Wireless Network
HetNet	Heterogeneous Network
HMIMO	Holographic MIMO
IM	Index Modulation
IoT	Internet of Things
IRS	Intelligent Reflecting Surface
ITU	International Telecommunication Union
IW	Intelligent Walls
LIMS	Large Intelligent Metasurface
LISA	Large Intelligent Surface Antenna
LIS	Large Intelligent Surface
LoS	Line of Sight
LTE	Long-term Evolution
M2M	Machine to Machine
MEC	Mobile Edge Computing
MEMS	Micro-Electromechanical System
mmWave	Millimeter Wave
MIMO	Multiple-Input Multiple-Output
mMIMO	massive Multiple Input and Multiple Output
MM	Metamaterials
MMTC	Massive Machine-Type Communications
MS	Metasurface
MU	MultiUser
NP	Nondeterministic Polynomial-time
NLoS	Non Line of Sight
OFDMA	Orthogonal Frequency Division Multiplexing
PIN	Positive-Intrinsic-Negative
PIS	Passive Intelligent Surface
QoS	Quality of Service
RF	Radio Frequency
RFID	Radio Frequency IDentifier
RIS	Reconfigurable Intelligent Surface
SDMS	Software Defined Meta-Surface
SE	Spectrum Efficiency
SINR	Signal-to-Interference plus Noise Ratio
SNR	Signal-to-Noise Ratio
SM	Spatial Modulation
SMM	Spatial Microwave Modulators
UAV	Unmanned Aerial Vehicle
URLLC	Ultrareliable Low Latency Communication
VR	Virtual Reality
